# Supplementary material for: TOMM40 RNA Transcription in Alzheimer’s Disease Brain and Its Implication in Mitochondrial Dysfunction
Source: Genes (Basel). 2021 Jun 6;12(6):871. doi: 10.3390/genes12060871 (PMC8226536; doi:10.3390/genes12060871)
Supplement: Supplementary file 1 [file genes-12-00871-s001.zip › genes-1241487-supplementary.pdf]

Supplementary Figures and Tables

Figure S1. Allele-specific primers of *TOMM40* pseudogenes can cross-amplify other pseudogenes.

Figure S2. Conventional *TOMM40* cDNA assays can cross-amplify *TOMM40* pseudogenes.

Table S1. Primers, probes, and TaqMan assays.

Table S2. Fractions of *TOMM40* pseudogene *P1b/P2* RNAs and IVS9 measured RNA in total *TOMM40*-related RNA pool, measured by dPCR.

Figure S1. Allele-specific primers of *TOMM40* pseudogenes can cross-amplify other pseudogenes. Capillary gel electrophoresis images of the end-point PCR amplified pseudogene (PG) amplicons. The P1 primer set cross-amplified all pseudogene templates (lanes 3-5) and *TOMM40* cDNA (lane 6). The P1b/P2 primer set cross-amplified templates of P3 (lane 4) and P4 (lane 5). The P3 primer set cross-amplified templates of P1b/P2 (lane 3) and P4 (lane 5). The P4 primer set cross-amplified P1b/P2 template (lane 3). Genomic DNA (lane 1) served as a positive control and no-template control (NTC, lane 2) served as a negative control.

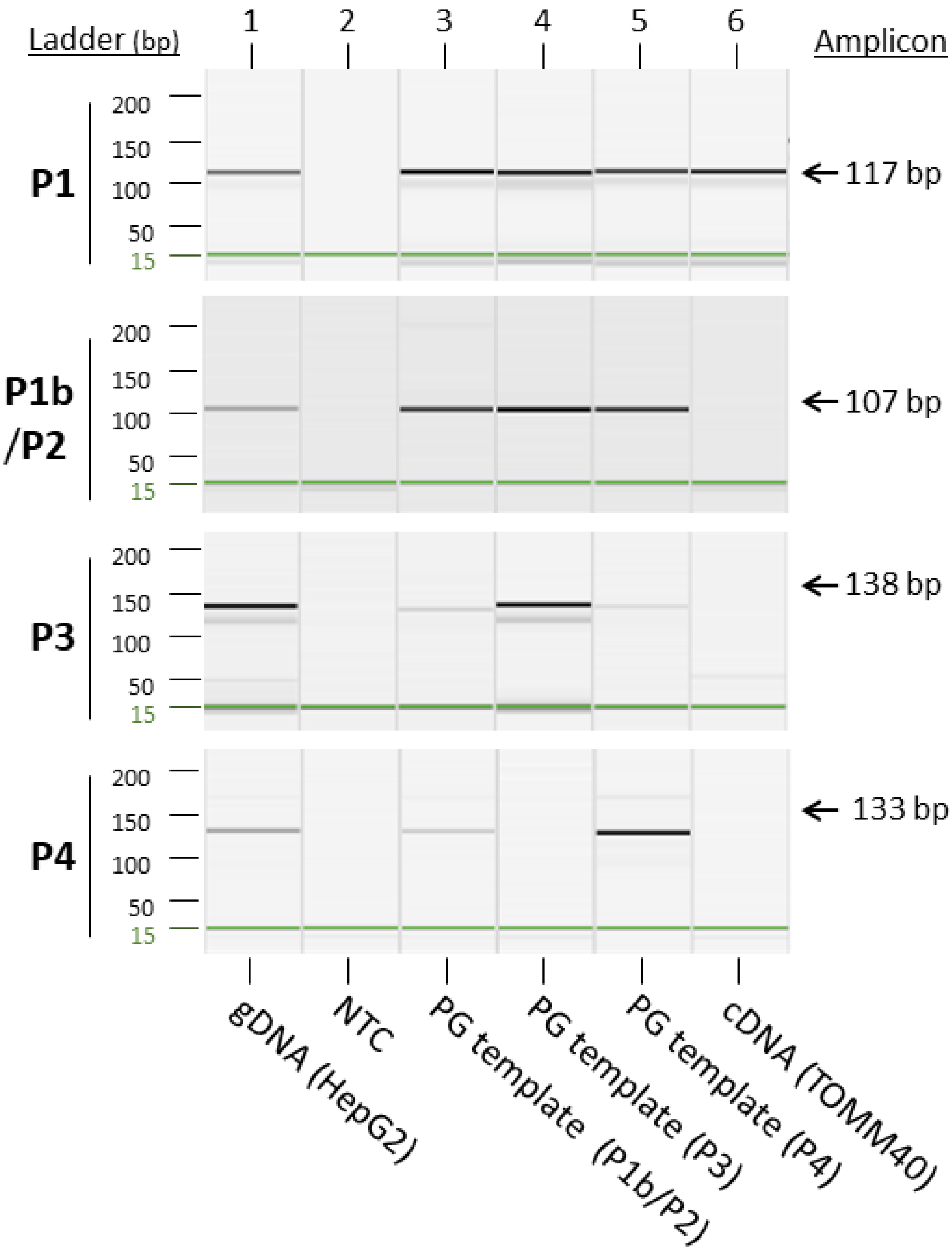

Figure S2. Conventional *TOMM40* cDNA assays can cross-amplify *TOMM40* pseudogenes. Capillary gel electrophoresis images of the end-point PCR amplified pseudogene (PG) amplicons. The spliced mRNA-based cDNA assays span the splicing junctions of either exons 1 and 2 (Ex1-Ex2) or exons 3 and 4 (Ex3-Ex4) of *TOMM40*. RT-PCR amplified *TOMM40* amplicons from three cell lines (HepG2, U-87, SH-SY5Y; lanes 1-3) and *TOMM40* cDNA (lane 7) are shown with the expected size. These two assays also amplified all the DNA templates of *TOMM40* pseudogenes from (lanes 4-6) with the expected size of amplicons. No-template control (NTC, lane 8) served as a negative control.

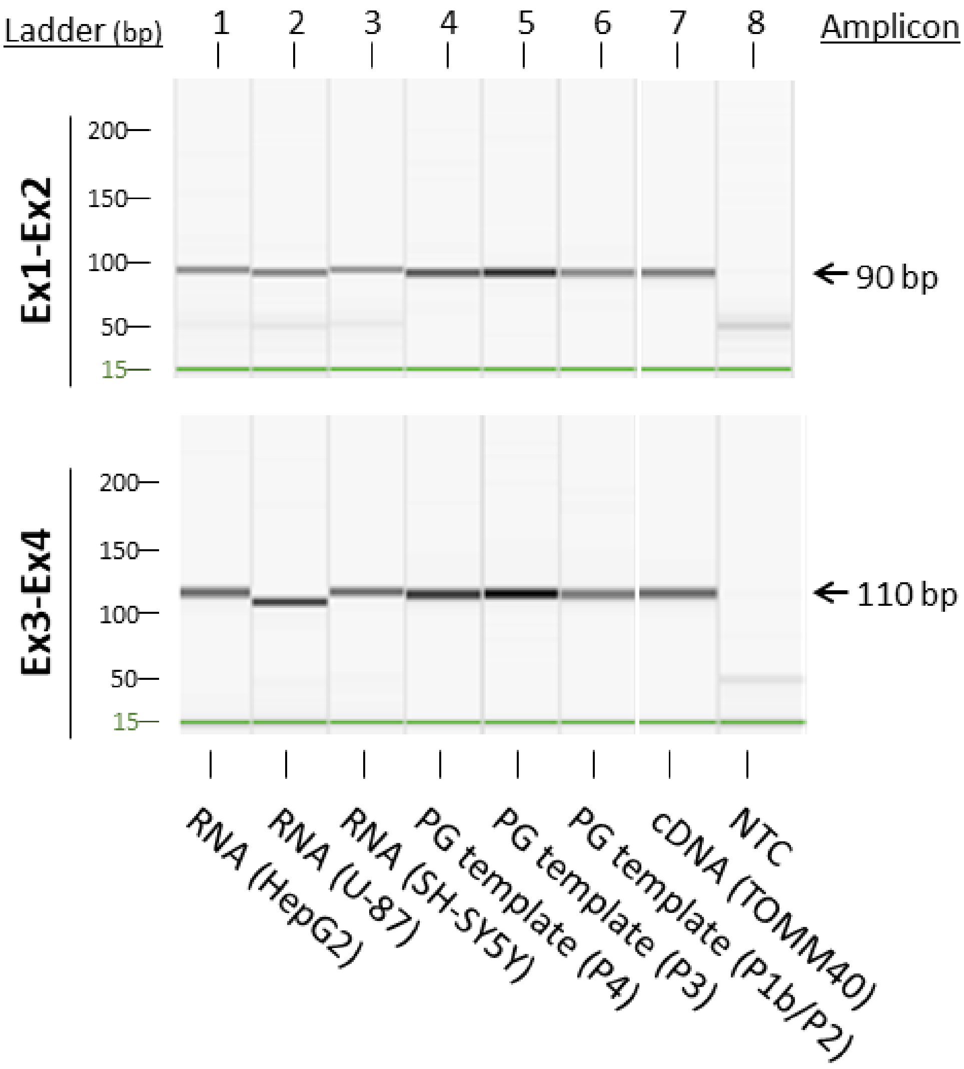

Table S1. Primers, probes, and TaqMan assays.

Usage	Primer Name	Sequence 5'-	Amplicon (bp)	Comment
**TOMM40 pseudogene template**				
	PG_P1b/P2_F1	TAACCCAAACAAGCAAAACATTAGT	1901	Forward primer for TOMM40P1b, P2, and P4
	PG_P1b/P2_R1	GAGCATTTTATCAAATTCCCCTAGT		Reverse primer for TOMM40P1b and P2
	PG_P3_F1	TGGACATGTCACATTAAGACAGACT	1887	Forward primer for TOMM40P3
	PG_P3_R1	TGTTCCACACTCTCTGAACTAACTG		Reverse primer for TOMM40P3
	PG_P4_R1	CTAAAAGCCTTAGATAAGCATGGTG	1770	Reverse primer for TOMM40P4
**TOMM40 pseudogene RNA**				
	T40-PG_P1_F1	GAGTGCCACCGGAGGTGTAAGG	117	Forward primer for TOMM40P1
	T40-PG_P1_R1	GGCTACTGTGTGGTTCACCTGG		Reverse primer for TOMM40P1
	T40-PG_P1b/P2_F1	GTTCTTGCGGTGATTCAGGAAGGC	107	Forward primer for TOMM40P1b and P2
	T40-PG_P1b/P2_R1	CTCCTCTTCAAAGGCTCTGTGGATAGC		Reverse primer for TOMM40P1b and P2
	T40-PG_P3_F1	TTGCTTGGCCCTGGGCAGTG	138	Forward primer for TOMM40P3
	T40-PG_P3_R1	GGGTTTGTGGTGTTATGTCCAACCC		Reverse primer for TOMM40P3
	T40-PG_P4_F1	TCCTGAACCCACGAGGACGTC	133	Forward primer for TOMM40P4
	T40-PG_P4_R1	CCTCAGGTCCAAGATGGCCATCC		Reverse primer for TOMM40P4
**TOMM40 cDNA template**				
	T40_cDNA_F1	CACGGGGTGGGAGCGGAG	1275	Forward primer
	T40_cDNA_R1	TCCCTCCTTTCCAATGTCCC		Reverse primer
**TOMM40 mRNA**				
	T40_Ex1-Ex2_F1	ACCATGGGGAACGTGTTGGCTGC	90	Forward primer for TOMM0 Ex1-Ex2
	T40_Ex1-Ex2_R1	CGGAGGTGGCGGCAGCCCCACG		Reverse primer for TOMM0 Ex1-Ex2
	T40_Ex3-Ex4_F1	CAAAGGGTTGAGTAACCATTTTCAGGTCAACCAC	110	Forward primer for TOMM40 Ex3-Ex4
	T40_Ex3-Ex4_R1	GACTCAGCTGCTTTGTCCCCACATATGTG		Reverse primer for TOMM40 Ex3-Ex4
	T40_Ex4-Ex5_F1	TCAGCACAATCGGGGAGTC	112	Forward primer for TOMM0 Ex4-Ex5
	T40_Ex4-Ex5_R1	CCACTGTTGTCCATGTCACCC		Reverse primer for TOMM0 Ex4-Ex5
	TOMM40 TaqMan GX	N/A		Thermo Fisher (Hs01587378_mH)
	ACTB TaqMan GX	N/A		Thermo Fisher (Hs01060665_g1)
**TOMM40 primary RNA transcript**				
	T40_Ex6-IVS6_F1	CTCGTGGGTTCAGGTAAGA	98	Forward primer for TOMM40 surrogate Ex6-IVS6
	T40_Ex6-IVS6_R1	AAAGGAGGTTCACAAATGGG		Reverse primer for TOMM40 surrogate Ex6-IVS6
	T40_Ex6-IVS6 Probe	FAM-ATTTGCTGGCAAAGCCAGAACTGC-IBFQ		Ordered from Integrated DNA Technologies
	T40_IVS9_F1	GCAGGAGCAGTGTGGTTAAA	85	Forward primer for TOMM40 surrogate IVS9
	T40_IVS9_R1	CACCATATCCATGCAGAGTTCC		Reverse primer for TOMM40 surrogate IVS9
	T40_IVS9_Probe	FAM-TCGGCATGTGGCTGGTATCCAA-IBFQ		Ordered from Integrated DNA Technologies
**Mitochondrial DNA copy number**				
	HsMt_15977_F1	CCACCATTAGCACCCAAAGCT	115	Forward primer
	HsMt_16091_R1	TACATAGCGGTTGTTGATGGG		Reverse primer
**Hemoglobin B for MtDNA copy number normalization**				
	HGB_F1	TGTGCTGGCCCATCACTTTG	73	Forward primer
	HGB_R1	ACCAGCCACCACTTTCTGATAGG		Reverse primer

Table S2. Fractions of *TOMM40* pseudogene *P1b/P2* RNAs and IVS9 RNA in total *TOMM40*-related RNA pool, measured by dPCR.

PMB_ID	Copies/µl (95% Poisson confidence intervals)			% in Total *TOMM40*	
	Total *TOMM40*	*P1b/P2*	IVS9	*P1b/P2*	IVS9
AD_1	55.9 (3.3)	11.6 (1.5)	12.2 (1.6)	20.8	21.8
AD_2	76.7 (3.8)	15.2 (1.7)	11.5 (1.5)	19.8	15.0
AD_3	201.4 (6)	22.3 (2)	7.9 (1.2)	11.1	3.9
AD_4	96.7 (4.2)	14.4 (1.6)	16 (1.7)	14.9	16.5
AD_5	145.9 (5.3)	32.9 (2.5)	11.6 (1.6)	22.5	8.0
AD_6	101.8 (4.4)	16.9 (1.8)	16.8 (1.9)	16.6	16.5
AD_7	205.3 (8.2)	23.6 (2.1)	25.2 (2.6)	11.5	12.3
AD_8	65.8 (3.6)	7.9 (1.3)	18.1 (2.2)	12.0	27.5
AD_9	107.7 (4.6)	15.7 (1.7)	30.9 (2.9)	14.6	28.7
AD_10	155.9 (5.5)	13.2 (1.6)	51.2 (3.7)	8.5	32.8
AD_11	82.4 (4.3)	14.7 (1.7)	12.1 (1.6)	17.8	14.7
AD_12	127.2 (4.8)	29.8 (2.3)	14.5 (1.7)	23.4	11.4
AD_13	242 (6.8)	44.2 (2.8)	18.4 (1.9)	18.3	7.6
AD_14	64.6 (3.4)	9.5 (1.3)	12.8 (1.5)	14.7	19.8
AD_15	206.5 (6.4)	40.5 (2.8)	18.5 (2.2)	19.6	9.0
AD_16	117.2 (4.8)	16.6 (1.8)	10.1 (1.6)	14.2	8.6
AD_17	126.5 (4.9)	15.7 (1.7)	40.2 (3.3)	12.4	31.8
AD_18	59.5 (3.4)	9.5 (1.4)	9.8 (1.6)	16.0	16.5
AD_19	103.9 (4.5)	11.1 (1.5)	32.4 (2.6)	10.7	31.2
AD_20	110.7 (5.5)	15.8 (1.7)	11.8 (1.5)	14.3	10.7
AD_21	67.1 (3.6)	13.1 (1.6)	14.1 (1.7)	19.5	21.0
AD_22	126.7 (5.1)	12.4 (1.6)	17 (2.1)	9.8	13.4
AD_23	120.8 (5.6)	17 (1.8)	23.5 (2.5)	14.1	19.5
AD_24	89.2 (4.8)	13.5 (1.6)	7.1 (1.4)	15.1	8.0
AD_25	104.9 (5.2)	17.2 (1.8)	15.4 (2.1)	16.4	14.7
AD_26	261.8 (8.4)	40 (2.8)	15 (2)	15.3	5.7
AD_27	29.9 (2.8)	5.7 (1.1)	1.7 (0.7)	19.1	5.7
AD_28	142.8 (5.1)	33.5 (2.5)	17.3 (2.1)	23.5	12.1
AD_29	57.6 (3.4)	9.4 (1.4)	12.3 (1.6)	16.3	21.4
Ctrl_1	121.5 (4.9)	12.1 (1.6)	7.4 (1.2)	10.0	6.1
Ctrl_2	141.4 (5.2)	13.8 (1.6)	10.6 (1.7)	9.8	7.5
Ctrl_3	185.1 (5.9)	28.7 (2.4)	11.1 (1.5)	15.5	6.0
Ctrl_4	205.6 (6.4)	27.7 (2.3)	15.1 (1.7)	13.5	7.3
Ctrl_5	210.9 (6.1)	33.2 (2.5)	6 (1.1)	15.7	2.8
Ctrl_6	144.5 (5.1)	21.3 (2)	13.5 (1.6)	14.7	9.3
Ctrl_7	265.9 (7.2)	25.6 (2.2)	7.2 (1.2)	9.6	2.7
Ctrl_8	177.9 (5.9)	18 (1.9)	24.9 (2.3)	10.1	14.0
Ctrl_9	292 (7.6)	24.7 (2.2)	21.2 (2.4)	8.5	7.3
Ctrl_10	186.3 (6.1)	24.8 (2.2)	13.5 (1.9)	13.3	7.2
Ctrl_11	156.8 (5.3)	23.1 (2)	9.9 (1.4)	14.7	6.3
Ctrl_12	208.6 (6.3)	29.8 (2.4)	21.8 (2.1)	14.3	10.5
Ctrl_13	114 (4.8)	11.2 (1.5)	21.4 (2.1)	9.8	18.8
Ctrl_14	266.4 (7.5)	28.4 (2.4)	18.3 (2)	10.7	6.9
Ctrl_15	307.2 (8)	38.3 (2.7)	41.1 (3.4)	12.5	13.4
Ctrl_16	182 (6)	18.1 (1.9)	8.5 (1.5)	9.9	4.7
